# Antioxidant and Anti-Inflammatory Activities of a Natural Compound, Shizukahenriol, through Nrf2 Activation

**DOI:** 10.3390/molecules200915989

**Published:** 2015-09-02

**Authors:** Jong-Hyun Park, Ji Won Choi, Eun Ji Ju, Ae Nim Pae, Ki Duk Park

**Affiliations:** 1 Center for Neuro-Medicine, Korea Institute of Science and Technology, Seoul 136-791, Korea; E-Mails: 090749@kist.re.kr (J.-H.P.); 213507@kist.re.kr (J.W.C.); 113506@kist.re.kr (E.J.J.); anpae@kist.re.kr (A.N.P.); 2 Department of Biotechnology, Yonsei University, Seoul 120-749, Korea; 3 Department of Biological Chemistry, University of Science and Technology, Daejeon 305-350, Korea

**Keywords:** nuclear factor-E2-related factor 2, neuroprotection, BV-2 mouse microglial cells, natural products, shizukahenriol

## Abstract

Imbalance in the antioxidant defense system leads to detrimental consequences, such as neurological disorders. The Nrf2 signaling is known as a main pathway involved in cellular defense system. Nrf2 is a transcription factor that regulates oxidative stress response by inducing expression of various antioxidant enzyme genes. In this study, we screened several pure natural compounds for Nrf2 activator. Among them, shizukahenriol (SZH), isolated from *Chloranthus henryi*, activated Nrf2, and induced expression of the Nrf2-dependent antioxidant enzymes HO-1, GCLC, and GCLM in BV-2 microglial cells. This natural compound was also effective in suppressing production of inflammatory molecules NO, TNF-α, and inhibition of NF-κB p65 translocation to the nucleus in a dose-dependent manner. We also examined whether SZH rescued the microglial cells from oxidative stress-induced cell death. Pretreatment with SZH dose-dependently attenuated H_2_O_2_-induced cytotoxicity in BV-2 microglial cells. These results suggested SZH as a potential neuroprotective agent for neurological disorders.

## 1. Introduction

Neuroinflammation through microglial activation and oxidative stress have been believed to be involved in the pathogenesis of several neurodegenerative diseases including Parkinson’s disease (PD) and Alzheimer’s disease (AD) [1,2]. Oxidative stress is known to be caused by an imbalance between antioxidant defense systems and production of reactive oxygen species (ROS), such as hydrogen peroxide, nitric oxide, superoxide, and the highly reactive hydroxyl radicals [3,4]. Recent studies suggested that activation of the cellular defense system could be beneficial to survival of neuronal cells in neurological disorders [5,6,7,8].

The nuclear factor E2-related factor 2 (Nrf2) signaling pathway is mainly responsible for cellular defense against oxidative stress. The transcription factor Nrf2 regulates oxidative stress responses by inducing expression of antioxidant enzyme genes such as heme oxygenase 1 (HO-1), NAD(P)H quinone oxidoreductase 1 (NQO1), glutamate-cysteine ligase (GCL), which consists of both the modifier (GCLM) and catalytic (GCLC) subunits [9,10]. It has also been reported that Nrf2 activation down-regulates the NF-κB-associated inflammatory responses in macrophages and microglia [11,12,13,14]. NF-κB activation and microglial activation induced by LPS were attenuated by exposure to Nrf2 activators, such as sulforaphane and curcumin. [15,16].

We have recently reported that Nrf2 activation led to significant neuroprotection effects in dopaminergic neurons from cytotoxic damage in both *in vitro* and MPTP-induced *in vivo* models of PD [17]. In addition, Nrf2 activation led to an excellent anti-neuroinflammatory response both in microglia cell line and *in vivo* animal models of PD [18]. Among the established drugs, dimethyl fumarate (DMF, Tecfidera^®^, Biogen, Cambridge, MA, USA) is a well-known Nrf2 activator for the treatment of patients with relapsing forms of multiple sclerosis (MS). DMF exhibits potent inhibitory activities against a pro-inflammatory response in *in vitro* models of brain inflammation through the Nrf2 signaling pathway [19,20]. These results suggest that agents with both anti-inflammatory and antioxidant properties will have a great potential in PD therapy.

In this study, we further extended our study to look for a natural compound with such properties in microglia. Natural products and related structural scaffolds have been major contributors to drug development and have been shown to offer protective effects against oxidative stress not only by scavenging ROS, but also by inducing expression of cellular defense enzymes via the Nrf2 pathway [21,22,23]. In the course of screening for natural compounds activating Nrf2, we found a dimeric sesquiterpene compound, shizukahenriol (SZH), isolated from *Chloranthus henryi*, which is a perennial herb widely distributed in China [24] and Taiwan [25]. Several sesquiterpenoids and ditepenoids were isolated from *C. henryi* and examined for various biological activities, such as antitumor and tyrosinase inhibition [26,27,28]. Our previous work described the isolation and elucidation of SZH and its biological activities [29]. In this study, we evaluated it for its ability in activating Nrf2 and inducing various antioxidant enzyme actions, and suppressing expression of inflammatory molecules in activated microglia. In addition, SZH was tested for its ability to rescue the microglial cells from oxidative stress-induced cell death.

## 2. Results and Discussion

### 2.1. Extraction and Isolation of SZH

**Figure 1:**
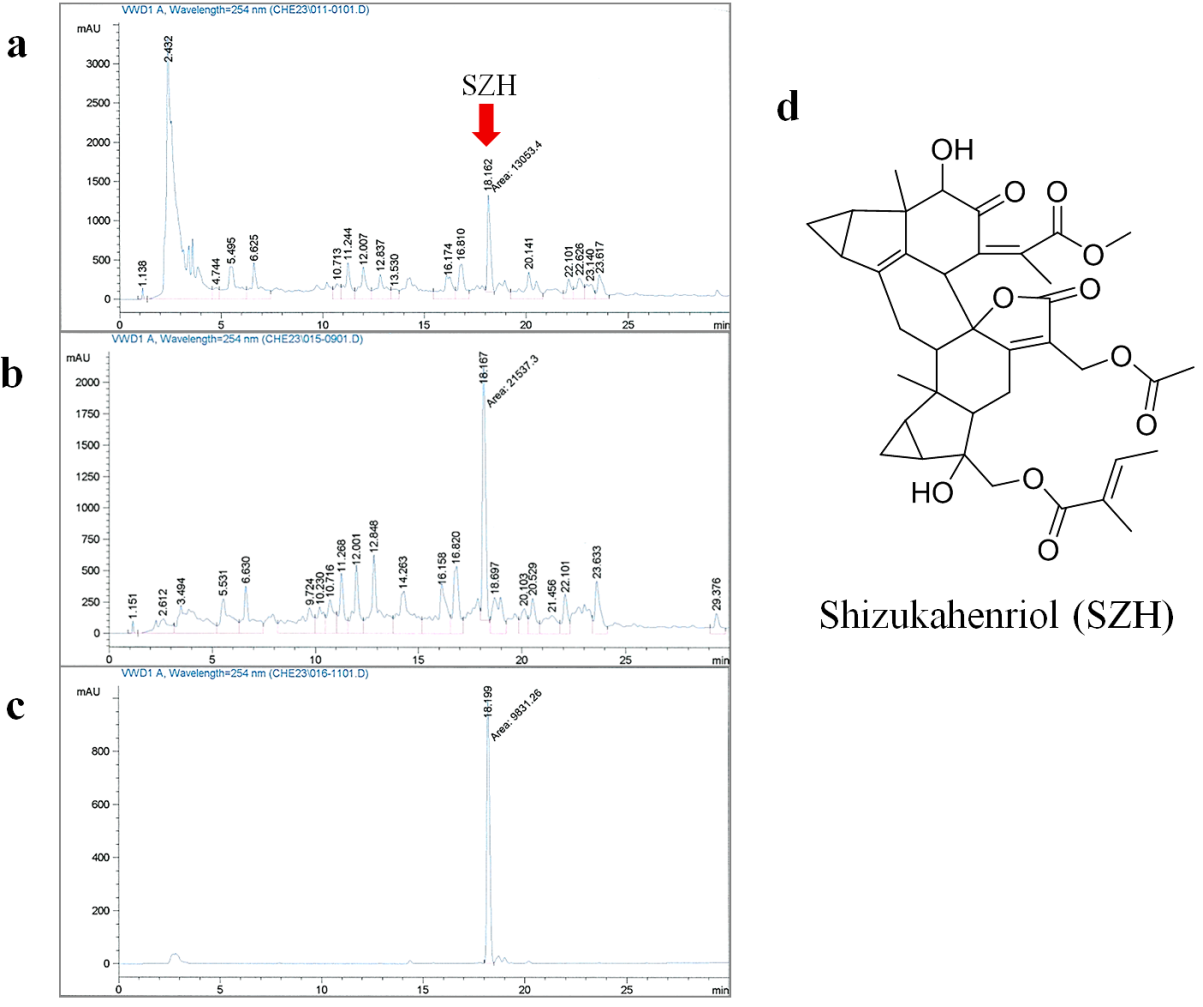
Extraction and isolation of SZH (**a**) HPLC analysis of SZH content after extraction with MeOH at room temperature for 72 h; (**b**) HPLC analysis of SZH content after extraction with optimized condition (EtOAc at 45 °C for 18 h); (**c**) HPLC analysis of the isolated compound; and (**d**) chemical structure of SZH.

In our previous study, the compound was isolated from either the methanol extract or MeOH/CHCl_3_ extract of *C. henryi* using various chromatography methods. In order to increase the isolation efficiency, we optimized and simplified the purification steps. EtOAc extraction led to a significant increase of SZH content and reduction of polar components (Figure 1a,b). The remaining polar compounds were removed with H_2_O washing three times, and then the resulting residue was chromatographed with silica gel to give a fairly pure crude compound. The crude compound was crystallized using EtOAc/*n*-hexane (4/1) with high yield (overall yield: 0.18%). The crystallized compound was confirmed as a single peak by analytical HPLC (Figure 1c) and characterized by various analytical instruments as SZH (Figure 1d).

### 2.2. Nrf2 Activation and Nrf2 Nuclear Translocation

**Figure 2:**
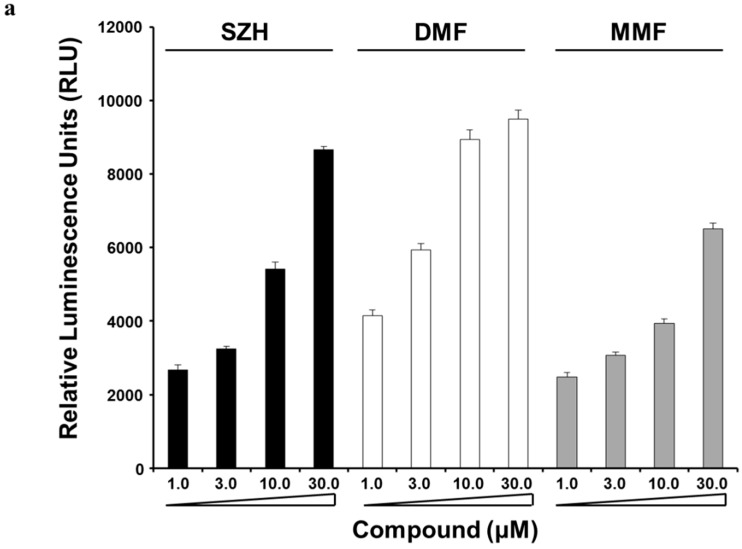

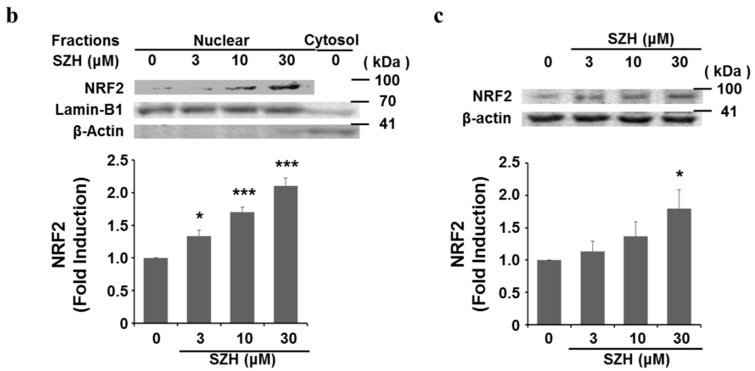
Effect of SZH on Nrf2 nuclear translocation (**a**) modified U2OS cells were treated with SZH, dimethyl fumarate (DMF) or monomethyl fumarate (MMF) in indicated concentration for 6 h and measured for RLU evoked by nuclear localization of Nrf2 in the PathHunter^®^ (DiscoveRx, Fremont, CA, USA) Keap1-Nrf2 functional assay system. Data values and error bars were performed in quadruple, and represent the mean ± S.E.M.; (**b**) BV-2 microglial cells were treated with various concentration of SZH for 24 h and nuclear extracts were prepared to analyze translocated Nrf2 level by western blots. Lamin-B1 and β-actin represented as nuclear fraction and cytosol fraction respectively; and (**c**) in a parallel experiment, Western blot showed total Nrf2 level of cell lysate. These blots were representatives of three independent experiments. Data were presented as the mean value of three experiments ± S.E.M. * *p* < 0.05 or *** *p* < 0.005 *vs.* DMSO only treated control.

Initially, we primarily screened for Nrf2 activators from pure natural compounds and evaluated the effect of SZH on Nrf2 activation by PathHunter^®^ Assay Complete U2OS cell culture Keap1-Nrf2 assay. Several results showed that Nrf2 is ubiquitously expressed in various organs as a transcriptional activator for phase II antioxidant enzyme genes by joining antioxidant responsive element (ARE) sequences [30]. As shown in Figure 2a, we demonstrated that SZH is a potent activator of Nrf2 release from Keap1 with elevated luminescence in a concentration-dependent manner, due to nuclear translocation of Nrf2. Recent studies have proposed that fumaric acid esters such as dimethyl fumarate (DMF) and its primary metabolite monomethyl fumarate (MMF) can activate genes associated with the Nrf2 antioxidant response pathway [20,31]. Therefore, we evaluated the effect of SZH on Nrf2 activation compared with DMF and MMF, and found that SZH exerted similar Nrf2 activation efficacy to DMF at 30 μM. SZH showed higher efficacy than MMF and lower efficacy than DMF at 10 μM (Figure 2a).

We also assessed the effect of SZH on Nrf2 activation via nuclear enrichment of Nrf2 in BV-2 microglial cells. Western blot data indicated that SZH dose-dependently increased Nrf2 abundance in the nucleus (Figure 2b). The fidelity of the nuclear preparations was confirmed by Western blot for nuclear membrane protein Lamin B1. Under homeostatic conditions, the Keap1-Cul3-E3 ubiquitin ligase complex tightly regulates low levels of Nrf2 via ubiquitin-mediated proteolysis [32]. Indeed, SZH resulted in elevation of total cellular Nrf2 protein levels (Figure 2c). These results implied that SZH caused Nrf2 activation by its translocation from the cytoplasm to the nucleus. 

### 2.3. SZH Induces Gene Expression of Antioxidant Enzymes in BV-2 Microglial Cells

**Figure 3:**
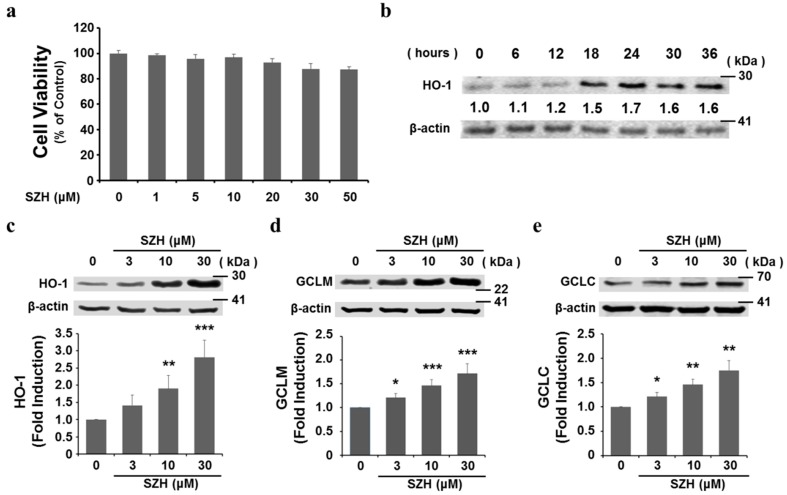
Induction of antioxidant enzyme gene expression by SZH in BV-2 microglial cells (**a**) effect of SZH on BV-2 microglial cell viability using CCK-8 cytotoxicity assay for 24 h; (**b**) expression kinetics of HO-1, a major nrf2-inducing enzyme, by SZH treatment; (**c**–**e**) BV-2 microglial cells were harvested after 24 h, the cell lysate were subjected to Western blot; (**c**) HO-1 (**d**) GCLM; and (**e**) GCLC, densitometric analyses were performed and the data were normalized against the internal control β-actin. These data are expressed as fold induction of untreated control ± S.E.M. * *p* < 0.05, ** *p* < 0.01 or *** *p* < 0.005 *vs.* DMSO only treated control.

To investigate the cytotoxic potential of SZH on BV-2 microglial cells, cells were incubated with different concentrations of SZH for 24 h and the cell viability was tested by CCK-8. The cells exposed to SZH alone did not cause significant changes in cell viability (Figure 3a). After nuclear translocation, Nrf2 subsequently binds to ARE sites in promoter regions of genes encoding phase II detoxifying and antioxidant enzymes [5]. We hypothesized that these enzyme genes might be induced in microglial cells by SZH in the same concentration range. At a concentration of 10 μM, HO-1 expression was detected early time point, the maximum increase was observed around 24 h in BV-2 microglial cells (Figure 3b). At 24 h, the protein level of HO-1, a major Nrf2-dependent enzyme that converts haem to biliverdin and carbon monoxide (CO), was increased by SZH in a dose-dependent manner (Figure 3c). GCL is also an Nrf2-dependent enzyme in the biosynthetic pathway for the major cellular antioxidant glutathione and consists of two subunits, the modulatory (GCLM) and catalytic (GCLC) subunits. We found that both GCLM and GCLC were also effectively induced by SZH (Figure 3d,e). These data denoted that Nrf2 migrated into the nucleus by SZH, and regulated the transcription of a battery of downstream genes that protect against cellular damage.

### 2.4. Effects of Shizukahenriol on LPS-Stimulated BV-2 Microglial Cells

**Figure 4:**
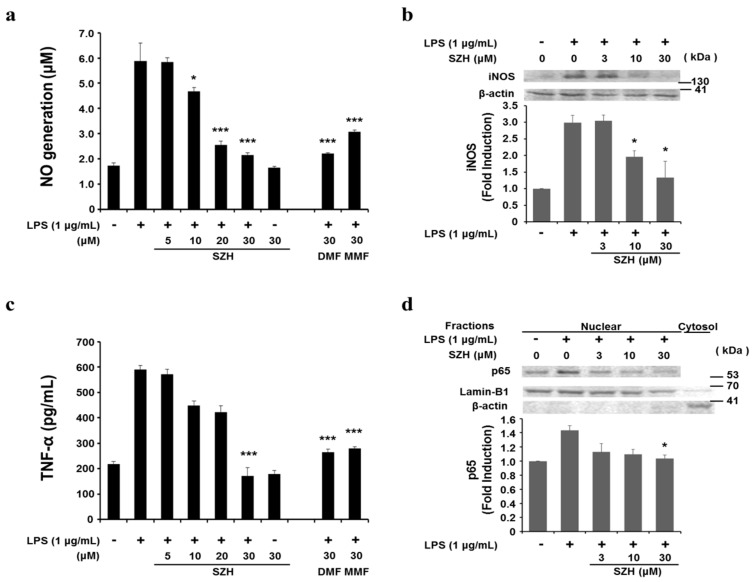
Attenuation of LPS-induced inflammation by SZH in BV-2 microglial cells (**a**) NO discharged into the culture medium was determined using the Griess reagent assay; (**b**) iNOS protein expression were calculated by Western blot analysis at 24 h; (**c**) the amounts of TNF-α in the supernatant were determined by ELISA; (**d**) NF-κB p65 in the nuclear fraction was estimated by Western blot analysis. After densitometry, the data were normalized against (**b**) β-actin or (**d**) Lamin-B1. Similar results were obtained in two additional immunoblots. Data are expressed as fold change of untreated control ± S.E.M. * *p* < 0.05 or *** *p* < 0.005 *vs.* LPS treatment group.

Recent studies have shown that natural compounds, such as licochalcone E and sulforaphane, that reduce lipopolysaccharide (LPS)-induced inflammatory responses also diminished iNOS-mediated NO production via NF-κB inactivation [15]. Therefore, to determine whether SZH suppressed an inflammatory response, we examined anti-inflammatory effects of SZH on LPS-stimulated BV-2 microglial cells. LPS (1.0 μg/mL) stimulation resulted in an increase of NO release (5.7 ± 0.8 μM) compared with the untreated control (1.7 ± 0.1 μM). However, LPS-induced NO production was significantly attenuated by 3 h pre-treatment with SZH in a dose-dependent manner (5.8 ± 0.15 μM at 5 μM, 4.7 ± 0.15 μM at 10 μM, 2.5 ± 0.17 μM at 20 μM, and 2.2 ± 0.09 μM at 30 μM SZH). We found that both DMF and MMF treatment also led to significant decreases in NO production (2.2 ± 0.03 μM at 30 μM DMF and 3.1 ± 0.08 μM at 30 μM MMF) (Figure 4a). In addition, the expression of iNOS protein was up-regulated in LPS-stimulated BV-2 microglial cells after 24 h. However, the degree of expression was dramatically diminished by SZH pre-treatment (Figure 4b). These results indicated that SZH attenuated LPS-stimulated NO production in BV-2 microglial cells, accompanied by the transcriptional regulation of the iNOS gene.

Next, we validated the effects of SZH on pro-inflammatory cytokine secretion. BV-2 microglial cells were incubated with SZH (5, 10, 20 or 30 μM) in the absence or presence of LPS (1.0 μg/mL) for 24 h, and cytokine levels in the culture media were measured by ELISA. As shown in Figure 4c, TNF-α level was increased in the media of LPS stimulated BV-2 microglial cells, and this increase was significantly suppressed in a concentration-dependent manner by SZH treatment. Thus, the results proposed that SZH suppressed the production of pro-inflammatory cytokine responsible for the inflammatory process. We also observed significant down-regulation of TNF-α production at 30 μM DMF and MMF. To further investigate the mode of action that SZH inhibits inflammatory expression, we examined SZH’s effect on NF-κB p65 nuclear translocation. The LPS-induced translocation of NF-κB p65 was significantly attenuated by pretreating with SZH (Figure 4d). These outcomes implied that the inhibition of NF-κB activation by SZH might be related to a mechanism responsible for suppression of NO and pro-inflammatory cytokine in BV-2 microglial cells.

### 2.5. Protection of SZH from Oxidative Stress Induced by H_2_O_2_

**Figure 5:**
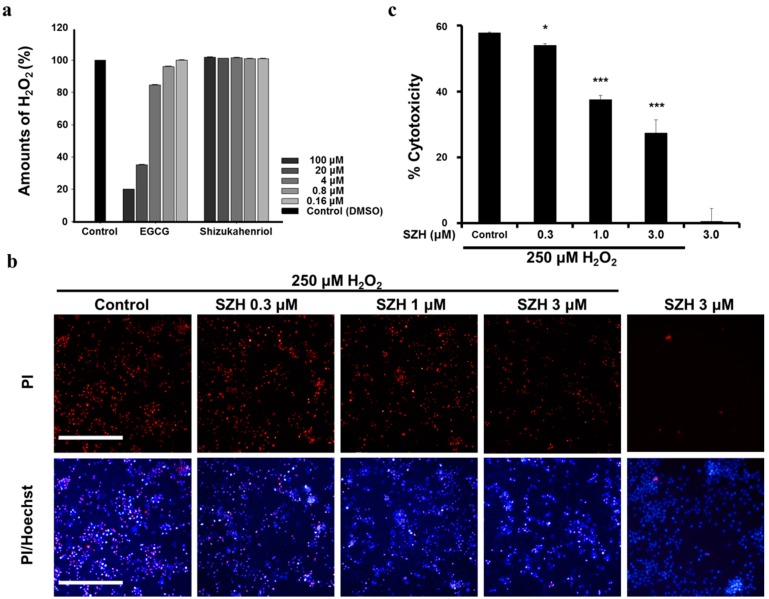
Protective effect of SZH in BV-2 microglial cells under H_2_O_2_ induced cytotoxicity (**a**) hydrogen peroxide scavenging activities of SZH; (**b**) the number of PI-stained nuclei was decreased with dose-dependent SZH treatment compared to vehicle treatment. The cells were pre-treated with SZH and exposed to 250 μM of H_2_O_2_ for 2 h. Fluorescent images were acquired by Operetta high content imaging system (10×, PerkinElmer, Waltham, MA, USA). PI-stained nuclei of dead cells were shown in red fluorescence and Hoechst-staining of all cells was shown in blue fluorescence; (**c**) H_2_O_2_ induced oxidative stress was quantified as a ratio of PI- and Hoechst-stained nuclei in BV-2 microglial cells. Bars represent means ± S.E.M. * *p* < 0.05 or *** *p* < 0.005 *vs.* H_2_O_2_ treatment control group.

Hydrogen peroxide (H_2_O_2_) is a highly reactive oxidant and induces oxidative stress. We evaluated protection effects of SZH against H_2_O_2_-induced cell cytotoxicity. First, we examined whether SZH directly exerted reactive oxygen species (ROS) scavenging activity. To determine the H_2_O_2_ scavenging activity of SZH, the H_2_O_2_ concentration was monitored by the modified Amplex red/horseradish peroxidase detection method. Figure 5a indicated that the H_2_O_2_ level was not changed by SZH itself in various concentrations (0.16 to 100 μM), whereas a positive control compound EGCG showed H_2_O_2_ scavenging activity in a dose-dependent manner.

We conducted experiments to evaluate the protective effects of SZH from the oxidative stress induced by H_2_O_2_ exposure in BV-2 microglial cells. SZH was dose-dependently pretreated overnight, following 2 h of 250 μM H_2_O_2_ incubation. PI fluorescent nucleic stain was treated in order to visualize cell death induced by H_2_O_2_. On the basis of fluorescence image results, we found that H_2_O_2_ exposure resulted in 60% cell death. The number of PI-stained nuclei was reduced in correlation with the dose-dependent treatment of SZH (Figure 5b). Furthermore, fluorescent nuclei were automatically analyzed and quantified from image results by the detection scheme in Harmony^®^ software (PerkinElmer, Waltham, MA, USA). The heat map was automatically generated and intensity of PI/Hoechst was dose-dependently decreased with SZH treatment. Pre-exposure to 3 μM SZH decreased 50% of cell cytotoxicity as compared to vehicle control (Figure 5c). In summary, SZH treatment led to the induction of the Nrf2-mediated anti-oxidative pathway, and the protection from oxidative injury. Interestingly, 3 μM SZH did not result in a significant increase in both the antioxidant and anti-inflammatory activities. It is possible that the combination of insignificant increases of both activities may have been sufficient in the cytoprotective activity against H_2_O_2_-induced cytotoxicity.

## 3. Experimental Section 

### 3.1. Extraction and Isolation of Shizukahenriol (SZH)

The dried stems and roots of *C. henryi* (200 g) were ground and extracted with EtOAc (1 L) at 45 °C for 18 h. The extract was filtered and washed with H_2_O (3 × 600 mL). The organic layer was dried over anhydrous Na_2_SO_4_ and concentrated in vacuum. The residue was purified by column chromatography on SiO_2_ (*n*-hexane/EtOAc 1/1). The desired fractions were collected and concentrated *in vacuo*. The resulting residue was crystallized (*n*-hexane/EtOAc 4/1) to give 0.2 g of shizukahenriol as a white solid. The purity of the compound was confirmed by HPLC. R*_f_* = 0.35 (*n*-hexane/EtOAc 1/1); mp: 173.8‒174.2 °C; ^1^H-NMR (400 MHz, DMSO) δ 0.20–0.67 (m, 2CHC**H**_2_, 2H), 0.79 (s, CHC**H_3_**, 3H), 0.91 (m, CHC**H**_2_, 1H), 0.93 (s, C**H_3_**, 3H), 1.12–1.16 (m, CHC**H**_2_, 1H), 1.36–1.39 (m, C**H**CH_2_, 1H), 1.55–1.56 (m, C**H**CH_2_, 1H), 1.59–1.64 (m, C**H**COH, 1H), 1.68 (d, *J* = 5.1 Hz, C**H**CH_2_, 1H), 1.72 (s, C**H_3_**C(O)OCH_3_, 3H), 1.80 (s, 2C**H**_3_, 6H), 1.88–1.91 (m, C**H**CH_2_, 2H), 2.03 (s, C(O)OC**H_3_**, 3H), 2.28–2.32 (m, C**H**_2_CHCOH, 1H), 2.38–2.42 (m, CHC**H**_2_, 1H), 2.66–2.71 (m, C**H**_2_CHCOH**,** 1H), 2.76 (d, *J* = 17.0 Hz, CHC**H**_2_, 1H), 3.60 (s, C(O)OC**H_3_**, 3H), 3.67(d, *J* = 11.4 Hz, C(O)OC**H**_2_COH, 1H), 3.72 (d, *J* = 5.0 Hz, C(O)C**H**COH, 1H), 3.90 (d, C(O)CC**H**, 1H), 4.10 (d, *J* = 11.4 Hz, CHC**H**_2_, 1H), 4.48 (s, C(O)OCH_2_O**H**, 1H), 4.62, 4.82 (d, *J* = 12.8, 12.9 Hz, C(O)OC**H_2_**, 2H), 5.77 (d, *J* = 5.1 Hz, C(O)CO**H**, 1H), 6.80–6.82 (m, CH_3_CC**H**CH_3_, 1H); ^13^C-NMR (400 MHz, CDCl_3_) *δ* 12.0 (CCH**C**H_2_), 12.2 (C(O)C**C**H_3_), 14.6 (CCH**C**H_3_), 15.3 (CCH**C**H_2_), 16.0 (C**C**H_3_), 20.4 (C(O)C**C**H_3_), 20.5 (C(O)**C**H_3_), 22.7 (C**C**H_2_), 24.8, 25.3 (2**C**HCH_2_), 25.4 (C**C**H_2_), 25.8 (**C**HCH_2_), 26.4 (C**C**H_3_), 28.4 (**C**HCOH), 41.0 (C(O)C**C**H), 44.8 (CCH3), 51.2 (**C**H_3_CCOH), 52.6 (**C**(O)OCH_3_), 55.1 (C(O)O**C**H_2_), 55.8, 60.5 (2CH_3_C**C**H), 70.9 (C(O)O**C**H_2_COH), 77.5 (CH**C**OH), 80.2 (C(O)**C**HOH), 93.3(C(O)O**C**CH), 123.7 (CH_2_**C**C(O)O), 128.1 (C(O)O**C**CH_3_), 131.6 (CH**C**CCH3), 132.0 (CH_3_**C**C(O)OCH_3_), 138.9 (C**C**HCH_3_), 142.3 (**C**CHCH_2_), 147.6 (**C**C(O)COH), 168.4 (**C**(O)OCH_2_), 170.4 (CH_3_**C**(O)OCH_2_), 170.5 (C**C**(O)OCH_3_), 171.4 (CH_2_C**C**(O)O), 172.2 (**C**CH_2_CH), 200.5 (CHC**C**(O)COH); HRMS [M + H]^+^ (ESI+) 677.2956 [M + H]^+^ (calcd for C_38_H_44_O_11_H + 677.2962).

### 3.2. Cell Culture

BV-2 mouse microglial cells were received from Onyou Hwang (University of Ulsan College of Medicine, Seoul, Korea) [18]. The cells were maintained in DMEM containing 10% FBS (Hyclone, Logan, UT, USA), 100 U/L penicillin and 100 μg/mL streptomycin (Gibco, Carlsbad, CA, USA) at 37 °C in 5% CO_2_ in a humidified atmosphere. For LPS-induced cell culture, cells were fed with fresh medium for treatment for 3 h prior to stimulation with LPS (1 μg/mL; *Escherichia coli* serotype O55:B5; Sigma-Aldrich, St. Louis, MO, USA) for 24 h.

### 3.3. Nrf2 Activation Assay

PathHunter^®^ AssayComplete™ U2OS Cell culture Keap1-Nrf2 assay (92-0018GL3, DiscoveRx, Fremont, CA, USA) was designed to measure a non-transcriptional response of activation-dependent Nrf2 translocation to the nucleus using a principal enzyme fragment complementation (EFC) technology. This EFC technology utilized a genetically engineered β-galactosidase enzyme consisting of two fragments—a large protein fragment (Enzyme Acceptor, EA) and a small peptide fragment (Enzyme Donor, ED). This action resulted in the complementation of the two β-galactosidase fragments, the activity of which is easily detected using chemiluminescent substrate with a microplate reader (SpectraMax^®^i3 multi-mode microplate reader; Molecular Device, Sunnyvale, CA, USA).

### 3.4. Cytotoxicity Assay

Cytotoxicity of SZH was evaluated in BV-2 microglial cells using a Cell Counting Kit-8 (CCK-8; Dojindo Molecular Technologies, Rockville, MD, USA). Cell viability was measured based on the formation of orange formazan metabolized from colorless WST-8 by mitochondrial dehydrogenases, which are active only in live cells. BV-2 microglial cells (15,000 cells/well) were seeded on 96-well plates and treated with various concentrations of SZH for 24 h. After 4 h treatment of CCK-8 reagent, the cell viability was determined by measuring the absorbance at 450 nm using a microplate reader (SpectraMax^®^i3; Molecular Devices). The relative measurement of cell viability for each treatment was calculated as compared to the absorbance of the control.

### 3.5. NO Assay

Concentrations of NO in the culture supernatants were determined by measuring nitrite, a major stable product of NO using the Griess reagent (sulfanilamide solution; 1% sulfanilamide in 5% phosphoric acid, NED solution; 0.1% *N*-1-napthylethylenediamine dihydrochloride in water). BV-2 cells (3.0 × 10^5^ cells/1.5 mL in 6 well plates) were treated with various concentrations of SZH with 1.0 μg/mL LPS for 24 h, and then 50 μL of each culture medium was mixed with an equal volume of Griess reagent. Nitrite levels were determined via microplate reader at 540 nm (SpectraMax^®^i3 multi-mode microplate reader; Molecular Device, Sunnyvale, CA, USA), and nitrite concentrations were calculated by reference to a standard curve generated by a known concentration of sodium nitrite (all reagents from Sigma-Aldrich, St. Louis, MO, USA).

### 3.6. Western Blotting

Cells were washed three times with PBS and lysed in RIPA lysis buffer (Sigma Aldrich) containing protease inhibitor cocktail tablets (Roche Diagnostics Corp., Indianapolis, IN, USA). Nuclear fractions were prepared using NE-PER nuclear extraction reagent (Pierce Biotechnology, Waltham, MA, USA) according to the manufacturer’s protocol. After centrifugation at 13,000 *g* for 20 min at 4 °C, the supernatant was obtained. Equal amounts (15 μg) of protein were separated on SDS-polyacrylamide minigels, transferred onto a nitrocellulose membrane (BIO-RAD, Munich, Germany) and blocked in 4% skim milk (Neogen, Lexington, KY, USA)-Tris-buffered saline Tween (TBST, 10 mM Tris, pH 7.5, 150 mM NaCl and 0.1% Tween 20) for 1 h at room temperature. The membranes were incubated overnight with primary antibody against NRF2 (D1Z9C; Cell signaling, Denvers, MA, USA, 1:300), HO-1 (Enzo Life Sciences, Ann Arbor, MI, USA, 1:1000), GCLC (Novus Biologicals, Littleton, CO, USA, 1:1000), GCLM (FL-274; Santa Cruz Biotechnology, Inc., Santa Cruz, CA, USA, 1:1000), iNOS (EPR16635; Abcam, Cambridge, UK, 1:500), NF-κB p65 (C22B4; Cell signaling, Denvers, MA, USA, 1:1000), LAMIN-B1 (D4Q4Z; Cell Signaling, 1:1000), and β-ACTIN (N-21; Santa Cruz Biotechnology, Inc., 1:1000) at 4 °C. Then, membranes were incubated for 1 h at room temperature with ScanLater EU-labeled secondary antibodies (Molecular Devices, Sunnyvale, CA, USA, 1:10,000 dilution in 4% skim milk). Protein bands were visualized using the SpectraMax^®^i3 ScanLater™ Western blot (Molecular Devices) and quantitatively analyzed by ImageJ software (version 1.49).

### 3.7. ELISA

BV-2 cells were seeded on six-well plates and treated with various concentrations of SZH for 3 h prior to stimulation with LPS (1 μg/mL, Sigma-Aldrich) for 24 h, and then the supernatant of culture medium was analyzed for secreted TNF-α by ELISA kit (eBioscience, San Diego, CA, USA) according to the manufacturer’s instructions. 

### 3.8. H_2_O_2_ Scavenging Capacity Test

To determine H_2_O_2_ scavenging capacity of the compound, H_2_O_2_ concentration was monitored using the modified Amplex red/horseradish peroxidase detection method [33]. The solution of H_2_O_2_ (200 μM, 50 μL) dissolved in assay buffer (50 mM phosphate buffer, pH 7.4) was treated with test compound (0.32–200 μM, 50 μL) and then was incubated for 10 min at room temperature. This reaction solution was added to assay solution (100 μL/well) containing 20 mM Amplex red (200 μL) and 200 U/mL HRP (100 μL) in assay buffer (9.5 mL) and protected from light. The absorbance measurement of the resulting solution was immediately performed with a microplate reader at 570 nm. The H_2_O_2_ scavenging capacities of the resulting solution were compared with control and EGCG (Epigallocatechin gallate) at various concentrations, well-known as a H_2_O_2_ scavenger.

### 3.9. Oxidative Stress in Vitro Assay Induced by H_2_O_2_

BV-2 cells were plated at a density of 5000 cells per well on 96-well microplates (Greiner Bio-One, Frickenhausen, Germany) and incubated for 24 h. Test compounds were dissolved in dimethylsulfoxide, (DMSO, final concentration < 0.5%) and diluted in culture media. The vehicle control was treated as identical concentration of DMSO. Compounds were pre-treated at various concentrations for 24 h. The media containing compounds were washed out and H_2_O_2_ diluted in serum-free media was treated for induction of oxidative stress. After incubation with 250 μM H_2_O_2_ for 2 h, cells were washed and incubated with culture media. Cytotoxicity of cells was measured by PI/Hoechst33342 co-staining. Cells were stained with 2 μg/mL Propidium Iodide (Sigma-Aldrich, excitation/emission of 535/617 nm) and 2 μg/mL Hoechst33342 (Thermo Scientific, Fisher, IL, USA, excitation/emission of 350 nm/461 nm) for 15 min. PI stains nuclei of dead cells, whereas Hoechst33342 stains nuclei of all cells. The fluorescent images were acquired by an Operetta high-content imaging system (Perkin Elmer, Waltham, MA, USA). The number of stained nuclei was automatically counted and H_2_O_2_-induced cytotoxicity was calculated as a ratio of number of PI-stained nuclei and number of Hoechst-stained nuclei (% cytotoxicity = # of PI-stained nuclei/# of Hoechst-stained nuclei × 100) by Harmony^®^ software. Merged images of PI/Hoechst stain indicated that purple stained (red + blue) nuclei represented dead cells whereas blue stained nuclei represented live cells.

### 3.10. Data Analysis

All data values and error bars were performed in triplicate, and represent the mean ± S.E.M. of three independent experiments. Statistical analysis of group differences was determined using an unpaired two-tailed Student’s *t*-test. * *p* < 0.05, ** *p* < 0.01, or *** *p* < 0.005 was considered statistically significant. 

## 4. Conclusions

In this study, the shizukahenriol (SZH), isolated from *Chloranthus henryi*, exhibited significant activities to induce expression of antioxidants and to suppress expression of pro-inflammatory enzymes through the Nrf2 pathway. The natural compound SZH significantly promoted the Nrf2 activating translocation and also elevated Nrf2 protein levels in the microglial cells. SZH was demonstrated to induce the expression of Nrf2-dependent antioxidant enzymes HO-1, GCLC, and GCLM. We also found that SZH could effectively down-regulate the production of inflammatory molecules NO, TNF-α, and NF-κB p65. In addition, pretreatment with SZH attenuated H_2_O_2_-induced cytotoxicity in BV-2 microglia cells in a dose-dependent manner.
